# Ensemble-Based Analysis of the Dynamic Allostery in the PSD-95 PDZ3 Domain in Relation to the General Variability of PDZ Structures

**DOI:** 10.3390/ijms21218348

**Published:** 2020-11-06

**Authors:** Dániel Dudola, Anett Hinsenkamp, Zoltán Gáspári

**Affiliations:** 1 Faculty of Information Technology and Bionics, Pázmány Péter Catholic University, Práter u. 50/A, 1083 Budapest, Hungary; daniel.dudola@gmail.com (D.D.); anetth@gmail.com (A.H.); 2 3in-PPCU Research Group, 2500 Esztergom, Hungary

**Keywords:** PDZ domain, internal dynamics, allostery, restrained molecular dynamics

## Abstract

PDZ domains are abundant interaction hubs found in a number of different proteins and they exhibit characteristic differences in their structure and ligand specificity. Their internal dynamics have been proposed to contribute to their biological activity *via* changes in conformational entropy upon ligand binding and allosteric modulation. Here we investigate dynamic structural ensembles of PDZ3 of the postsynaptic protein PSD-95, calculated based on previously published backbone and side-chain S${}^{2}$ order parameters. We show that there are distinct but interdependent structural rearrangements in PDZ3 upon ligand binding and the presence of the intramolecular allosteric modulator helix α3. We have also compared these rearrangements in PDZ1-2 of PSD-95 and the conformational diversity of an extended set of PDZ domains available in the PDB database. We conclude that although the opening-closing rearrangement, occurring upon ligand binding, is likely a general feature for all PDZ domains, the conformer redistribution upon ligand binding along this mode is domain-dependent. Our findings suggest that the structural and functional diversity of PDZ domains is accompanied by a diversity of internal motional modes and their interdependence.

## 1. Introduction

The postsynaptic density (PSD) of excitatory synapses is a well organized, dynamic network of proteins involved in processes of synaptic plasticity, learning and memory [1]. PSD-95 is the most abundant scaffold protein of the PSD taking part in a multitude of protein–protein interactions within the network [2]. It is involved in mechanisms/procedures including the stabilization, recruitment and trafficking of AMPA receptors to the postsynaptic membrane and dendritic spine morphogenesis during neurodevelopment, as well as in glutamatergic transmission and synaptic plasticity. As a MAGUK protein family member, PSD-95 contains 3 PDZ domains, an SH3 (Src homology 3) domain and a GK (guanylate kinase) domain. PSD-95 and related MAGUK proteins have been suggested to have two supramodular parts, the PDZ1-2 tandem and the PDZ3-SH3-GK domain unit. The PDZ1-2 tandem was shown to exhibit altered interdomain dynamics upon ligand binding [3]. In a recent computational study we have mapped the possible interdomain orientations in this supramodule and concluded that ligand binding modulates the structure and dynamics of the loop regions also participating in the interdomain interface, thereby suggesting a mechanistic link between intra- and interdomain dynamics [4]. The PDZ3-SH3-GK supramodule has also been shown to behave as a functionally coupled unit [5]. Models of the supertertiary structure of this supramodule suggest interactions between the PDZ3 and SH3 domains as well as a phopshorylation-dependent binding of a linker segment into the peptide binding groove of PDZ3 [6,7].

PDZ domains (named after three proteins in which they were first identified: PSD-95, Discs-large, ZO-1) are around 90 amino acid long globular modules consisting of five to six β-sheet and 2 α-helices in a conserved fold with a binding pocket between the second β-sheet (β2) and the second α-helix. PDZ domains typically interact with the C-terminal region of various peptides, but other forms of interactions are also known [8]. They are regularly classified according to the C-terminal sequences (last four residues) of their binding partners, although the specificity rules governing the interactions are more sophisticated. Structured extensions or additional elements (helices, loops, β-strands) can be found in a number of PDZ domains, which may influence binding. There are a number of studies aimed at identifying allosteric networks in PDZ domains, the results of which can vary widely depending on the methodology used [9]. Computational and experimental investigations on different domains conclude that although allostery is a general feature of PDZ domains, the exact residue networks are not necessarily the same and should be assessed separately for each case [10,11,12].

**Figure 1:**
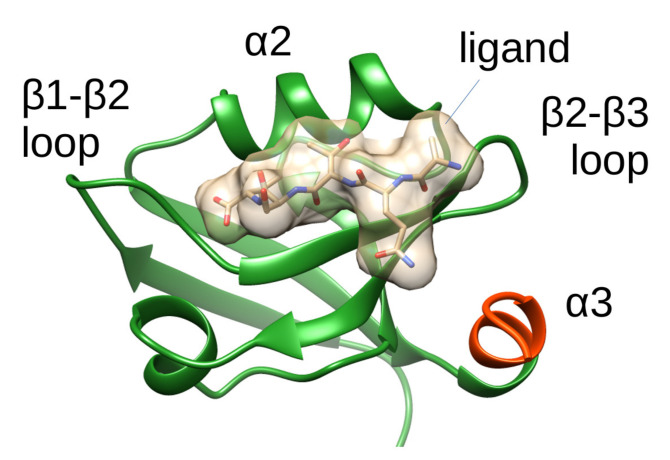
Ribbon representation of PSD-95 PDZ3 (residues 303-402) based on PDB structure 1BE9. The ligand, the α2 helix and the loops flanking the ligand binding site are labeled. The 7 residues of the additional α3 helix truncated in [21] are shown in red. The structure was drawn using UCSF Chimera.

PDZ3 of PSD-95 has been intensively studied both experimentally and by computational methods. It is a common subject of folding, evolutionary and mutational studies to assess the role of individual residues and interactions in ligand binding and allostery [13]. Statistical coupling analysis along with comprehensive mutational studies addressing the energetic coupling between residues identified generally different networks, although they all highlight the role of residues in strands β2, β3 and helix α2 [12,14,15,16,17,18,19,20]. Besides the common features of a PDZ domain fold, PDZ3 exhibits an additional C-terminal α-helical extension (termed α3 hereafter, Figure 1). Using NMR relaxation analysis, this extra helix was shown to act as an intramolecular allosteric modulator of ligand binding by comparing a construct bearing the full α3 helix with a variant lacking the last seven residues, termed Δ7CT. Truncation of the third helix leads to an over twentyfold decrease in the binding affinity, and the mechanism of this allosteric coupling was suggested to be of entropic origin by experiments [21]. Molecular dynamics calculations were qualitatively supporting this scenario, revealing changes in the flexibility of loops around the binding site [22]. Interaction of helix α3 with the rest of the domain is likely governed by electrostatic interactions, and the redistribution of these likely occurs upon ligand binding [23]. In addition, phosphorylation of Tyr397 of α3 alters the contacts of residues Glu331 and Glu334 in the β2-β3 loops with the ligand [24].

The role of loops in allosteric changes upon protonation of histidines and ligand binding has been reinforced by more recent analysis of μs-regime MD trajectories [25,26]. In some X-ray structures a succinimide ring was observed, formed by Asp 332 of the β2-β3 loop, although whether this modification occurs in vivo is not clear [27]. Mutational analysis of this residue revealed previously unobserved conformations of this loop when Asp332 was replaced with Gly [28].

Dynamic structural ensembles reflecting experimentally determined internal dynamics of proteins are valuable tools in investigating the functional aspects of structural transitions occurring at various time scales [29,30,31]. These ensembles serve as a structural interpretation of the parameters and can be produced with multiple methods, including restrained molecular dynamics. Such calculations use additional energy term(s) to ensure the compatibility of the structural ensemble with parameters like S${}^{2}$ order parameters or NOE distance restraints. S${}^{2}$ order parameters are usually obtained from NMR relaxation measurements and can be determined for protein backbone amide N-H and side-chain methyl groups [32]. These parameters report on the fast, ps-ns timescale dynamics of the given fragments and are ideal for restraining as they can be calculated from the atomic coordinates of a structural ensemble [33]. Restrained molecular dynamics using S${}^{2}$ order parameters usually relies on parallel calculation of replicas, and the restraining ensures that at any given time the simultaneously simulated replicas conform to the parameters [34]. Such calculations have been applied to the second PDZ domain of HPTP1E and revealed that the dynamical and evolutionary networks show only limited overlap [35].

In this study we integrate experimental data with computations, namely, using residue-level NMR-derived data on internal dynamics to generate conformational ensembles of four different states of PDZ3. We also provide an estimation of changes in conformational entropy between the free and bound states and analyze the structural differences between the states. With this approach we provide a detailed model of PDZ3 allostery that is consistent with experimental data and is based on shifting the equilibrium between well-defined states by the presence of both helix α3 and the ligand. To address the wider significance of the observed conformational transitions, we also performed a large-scale comparative analysis of available PDZ domains in the PDB, also including other ensembles either generated previously or specifically for this study.

## 2. Results

Dynamic structural ensembles were generated to model the free and peptide-bound states of the full and Δ7CT variant of PSD-95 PDZ3. The ensembles were verified for their compliance with experimental parameters and the observed changes in conformational entropy described in [21]. The conformational space explored by the different ensembles was analyzed in order to gain insight into the dynamical changes occurring upon ligand binding in the full and truncated forms.

### 2.1. The Calculated PDZ3 Ensembles Reflect Experimental Backbone and Side-Chain S${}^{2}$ Order Parameters

**Figure 2:**
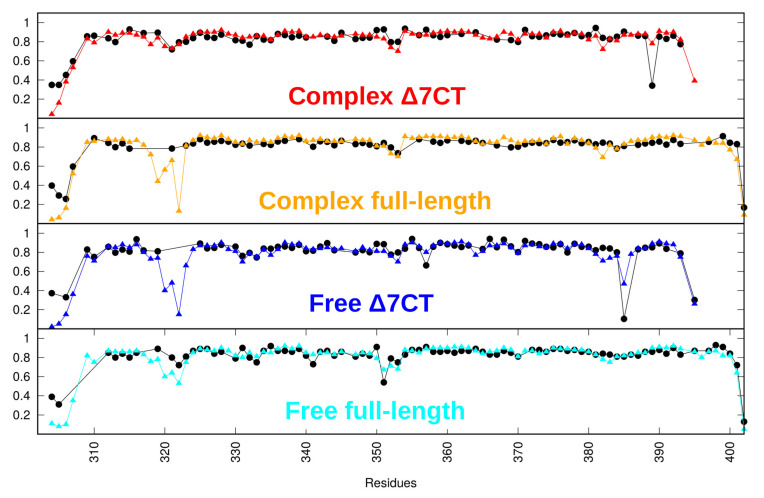
Measured (black) and back-calculated (colored according to key) backbone S${}^{2}$ values for the ensembles.

Implications of the side-chain dynamics having an apparent effect on the allostery [21,22] render it inevitable to include side-chain mobility in our ensemble-based models. To inspect the effects of side-chain order parameter restraining, exploratory simulations were carried out using different combinations of parameters available for PDZ3. Principal component analysis of ensembles generated with the only backbone, only side-chain and both S${}^{2}$ order parameters shows that the generated ensembles sample slightly different conformational ranges depending on the parameters used (Figure S1). Correlation with experimental order parameters was considerably lower for parameters not explicitly restrained for the particular ensembles (Table S1). Therefore, we decided to perform calculations with both backbone and side-chain S${}^{2}$ order parameters applied simultaneously, as they provide essential information to the ensembles. All our analysis and conclusions below refer to ensembles generated with both backbone and side-chain restraints (Figure 2, Table 1, Table S2).

### 2.2. Estimates of Changes in Conformational Entropy upon Binding Are Consistent with Experimental Observations

Conformational entropy of the PDZ3 ensembles was estimated using GROMACS with the quasi harmonic method [36] and the Schlitter formula [37]. It is not realistic to expect that the conformational entropy differences match the experimentally determined values, especially because different calculation methods, often considering different sets of atoms, yield substantially different estimates. It is also not expected that entropy values for larger ensembles—generated from longer simulations—show convergence regarding the absolute values. Our approach was to calculate the ratio of the binding entropy (defined as the difference between the free and complex structures) of the full-length and Δ7ct forms. The ratios calculated this way were checked for convergence.

In all cases, the free Δ7CT ensemble had the highest entropy, while the entropy values of the other states were comparable to each other. Therefore, the Δ7ct form exhibits a larger entropy change upon binding than the full-length domain. These observations are consistent with the findings in [21]. The ensembles derived from our 20 ns simulations have an entropy difference ratio $\left(S_{cd}-S_{fd}\right)/\left(S_{cf}-S_{ff}\right)$ of 4.1 that is considered to be in a reasonably good agreement with the experimental value of 2.4 obtained from isothermal titration calorimetry measurements reported in [21]. Thus, our ensembles reflect both experimental order parameters and conformational entropy changes well enough to be suitable for further detailed analysis.

### 2.3. Principal Component Analysis Reveals Distinct Motional Modes Affecting the Ligand Binding Site in PDZ3

**Figure 3:**
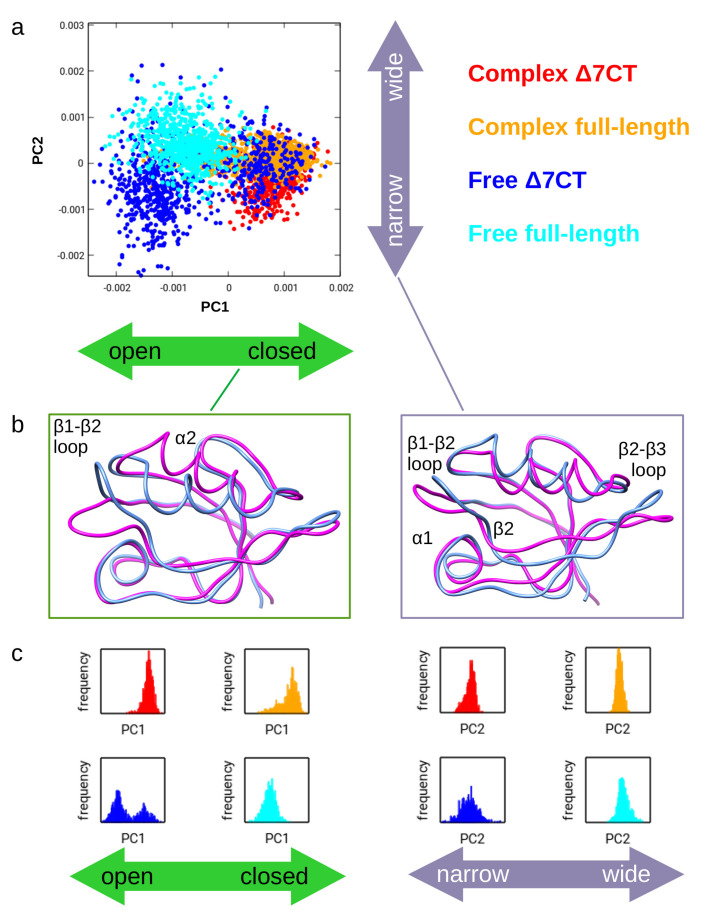
Principal component analysis of the combined ensembles for PSD-95 PDZ3. (**a**) Plot showing the distribution of conformers along the first two modes (PC1 and PC2). Colors represent the four ensembles as shown on the key on the right. PC1 is identified as an opening-closing motion and PC2 as a narrow-wide transition around the ligand binding site. (**b**) structural representation of the rearrangements corresponding to the identified motions. The two extremes of the motions are shown in magenta and cornflower blue (magnitudes of the motions are arbitrary for visualization purposes). Structures were rendered with UCSF Chimera. The key structural elements affected by the motions are labeled. (**c**) histograms showing the distribution of the conformers in the different ensembles along PC1 and PC2. Coloring is the same as in panel (**a**).

Principal component analysis (PCA) was carried for the combined ensemble of the four states. Only residues that are common to all variants are considered, thus, the C-terminal helix was omitted. Based on an exploratory analysis, the highly mobile N-terminal region was also truncated to be able to focus to motions in the structured core of the domains (residues 310–394). Principal component analysis of the combined ensemble reveals different types of motions around the ligand binding site (Figure 3).

The first mode, corresponding to principal component 1 (PC1) represents an opening-closing motion primarily affecting the β1-β2 loop and the C-terminal region of the α2 helix, which are in close proximity to each other and at the C-terminus of the ligand peptide. The second mode (PC2) affects the β1-β2 and β2-β3 loops and corresponds to a concerted displacement of the α2 helix and the β2 strand. We will refer to this mode as a narrow-wide transition. This mode also affects residues 293–294, close to α3, not explicitly considered in this analysis. The third mode affects multiple regions but mostly the β1-β2 and β2-β3 loops at opposite ends of the binding grove (Figure 3 and Figure S3).

As our parallel simulations comprised eight replicas, thus eight different trajectories, it is important to check whether replicas are “trapped” in the open and closed conformations of individual trajectories can visit both states. We have observed that the free Δ7CT form can fluctuate between these states within an individual trajectory, supporting that this type of motion is an inherent dynamic property of the domain. We have also compared our ensemble to the structures recently published by Camara-Artigas et al. [28] containing D332G and D332P mutants and showing variable conformations of the β2-β3 loop. We found that our ensemble covers the variability of these structures (Figure S4).

The first mode dominates the variability of the combined ensemble by accounting for almost 40% of its diversity covered by the first 10 modes (Figure S3). The ligand-bound structures populate primarily the closed conformation, whereas a substantial fraction of the unbound, free domains explore the open state. The Δ7CT structure, lacking the C-terminal “extra” helix α3, populates the open and closed states almost equally, whereas the unbound full domain remains in the open conformation. The narrow-wide difference seems to be affected by the presence of helix α3: full structures populate the narrow conformation and the Δ7CT variants the wide state, though the preferences are less pronounced than for the open-closed states (Table 2). Again, the free Δ7CT structure explores the largest region along this mode. Principal components and thus the motions they represent are by definition independent; nevertheless, this does not exclude that the states—defined as a region along one of the PCs—can preferably occur in specific combinations in some of the individual ensembles examined here. This is the case for the free full-length ensemble which almost exclusively occupies the open—wide region of the conformational space (Table 2).

### 2.4. Residue–Residue Contacts Affected by the Motions and the Presence of Helix α3 and the Ligand

**Figure 4:**
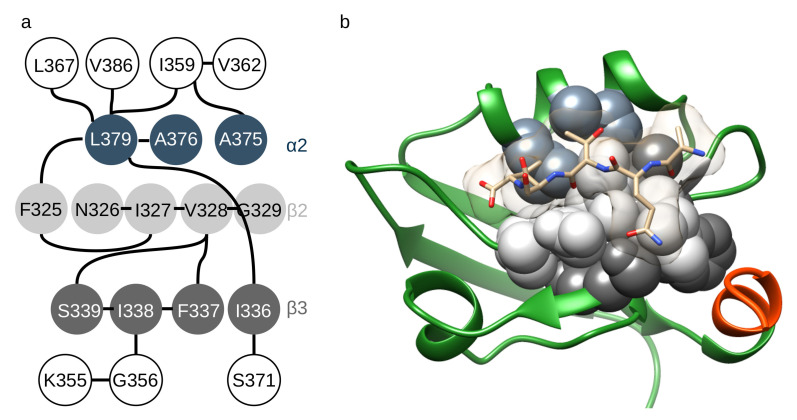
Residue contact network including contacts with discriminative power between two of the four possible states defined by ligand binding and presence of helix α3. (**a**) network displayed emphasizing the role of residues in β2, β3 or α2. (**b**) structural representation of the residues in the network and residing in the secondary structure elements mentioned (β2: light gray, β3: dark gray, α2: slate gray). Figure rendered with UCSF Chimera.

We have calculated residue–residue contacts for each structure in the ensembles and analyzed how changes in these contacts are associated with ligand binding and the presence of helix α3. The 28 contacts with discriminating power at least for one pair of states (see Methods) form a discontinuous graph, with one connected subgraph consisting of 19 residues as nodes. Of these, 12 have a nonzero betweenness centrality. We propose this network as one possible set of interactions responsible for allosteric communication (Figure 4). These residues connect strand β2 to β3 and both of these to helix α2. Residues 326–329 of strand β2, 339 of strand β3 as well as 376 and 379 of helix α2 are in contact with the ligand in the complexed ensembles. Residues 337–339 are also in direct contact with the residues 394–397 of the C-terminal helix α3.

### 2.5. Comparison of PSD-95 PDZ3 with an Extended Set of PDZ Domains

We intended to examine whether the structural rearrangements observed for PDZ3 are commonly found in other PDZ domains and how the structural diversity of a large set of PDZ domains relates to these. To this end, we compiled an extended set of PDZ structures from multiple sources. First, we have included the ensembles of PSD-95 PDZ1 and PDZ2 generated in our previous study [4]. Second, we selected small ensembles using the CoNSEnsX${}^{+}$ server from an MD-derived conformer pool for PDZ domains for which NMR chemical shifts are available (see Methods). Third, we included a number of PDZ domain structures as available in the PDB. The full set, including all three sources, is expected to sufficiently cover the conformational space occupied by different PDZ domains.

Comparison of a large set of domains is only possible if a common structural core is defined, and this core—the set of structurally corresponding residues—is smaller for larger structure sets. To identify the common residues in different sets of the domain analyzed, we have used multiple structural alignments and mapped the structures by retaining the Cα atoms of the residues in the core with a consensus numbering.

**Figure 5:**
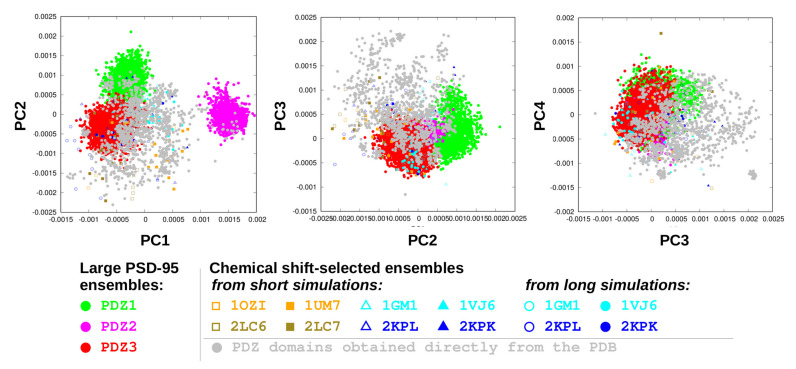
PCA analysis of the extended PDZ domain set.

Principal component analysis of the extended PDZ domain sets (Figure 5) reveals that the three PDZ domains of PSD-95 occupy different regions in the conformational space defined by the first two principal components. It should be noted that as in this analysis only residues in the common “PDZ core” are used, this difference is not a consequence of the presence of highly variable loop regions. In this context, PC1 corresponds to structural differences occurring mainly in the region connecting strand β6 with helix α2. Along PC2, the largest difference is the distance of β2 and α2, qualitatively reminiscent of the narrow-wide transition identified for PDZ3 above. PC3 describes a “seesaw” motion of α2 and PC4 resembles the opening-closing motion described above (see also Table 3 and more details below). PSD-95 PDZ2 seems to adopt a largely unique structure along PC1. Even in our full PDZ set (Figure 5b), only two structures can be found in or near the region defined by PDZ2. The first one is, not surprisingly, the second PDZ domain in the structure 2KA9 [3], the experimental NMR structure of the PDZ1-2 tandem of PSD-95 and the starting structure used to generate the PSD-95 PDZ2 ensemble [4]. The other structure is 1B8Q, corresponding to the PDZ domain of neuronal NO synthase, a protein that can interact with PSD-95. The interaction is between PDZ2 of PSD-95 and a segment located before the PDZ of nNOS [38].

While PCs 1 and 2 correspond to structural differences between different PDZ domains, in the PC3-PC4 plot, all structures form a largely homogeneous group, indicating that these components describe states that are accessible for most domains. Specifically, all three PSD-95 PDZ ensembles are largely overlapping in these modes. We provide a more detailed analysis of these modes later below.

### 2.6. The Three PDZ Domains of PSD-95

To analyze the differences between the PDZ domains of PSD-95, we compared the ensembles for these three domains using two approaches. In the first, we considered all resides that are common to these three domains, in the second, we used only the “PDZ core” common to all investigated structures as defined using a multiple structure alignment. Principal component analysis using these two approaches shows largely similar results, indicating that the differences between the domains are present regardless whether larger segments in the loop regions are included in the analysis or not. The PCA overlap analysis clearly shows that the first two modes capture essentially the same motions and the 3rd and 4th also describe highly similar motions. We have also analyzed the overlap between the modes in the individual ensembles and the combined ensemble. This analysis reveals that the first two modes that define the differences between the domains do not correspond to characteristic motions of the individual domains. In contrast, the third mode, as suggested above, largely corresponds to the opening-closing motion identified in PDZ3. This is also true for PDZ1 but not for PDZ2, confirming our previous notion that when analyzed separately, PDZ1 and PDZ2 exhibit distinct major structural rearrangements upon ligand binding [4]. This, however, as discussed below, does not mean that there are no common aspects of ligand-induced changes in the domains when analyzed as part of a larger dataset.

### 2.7. Comparison of the Free and Complexed States of Different PDZ Domains

**Figure 6:**
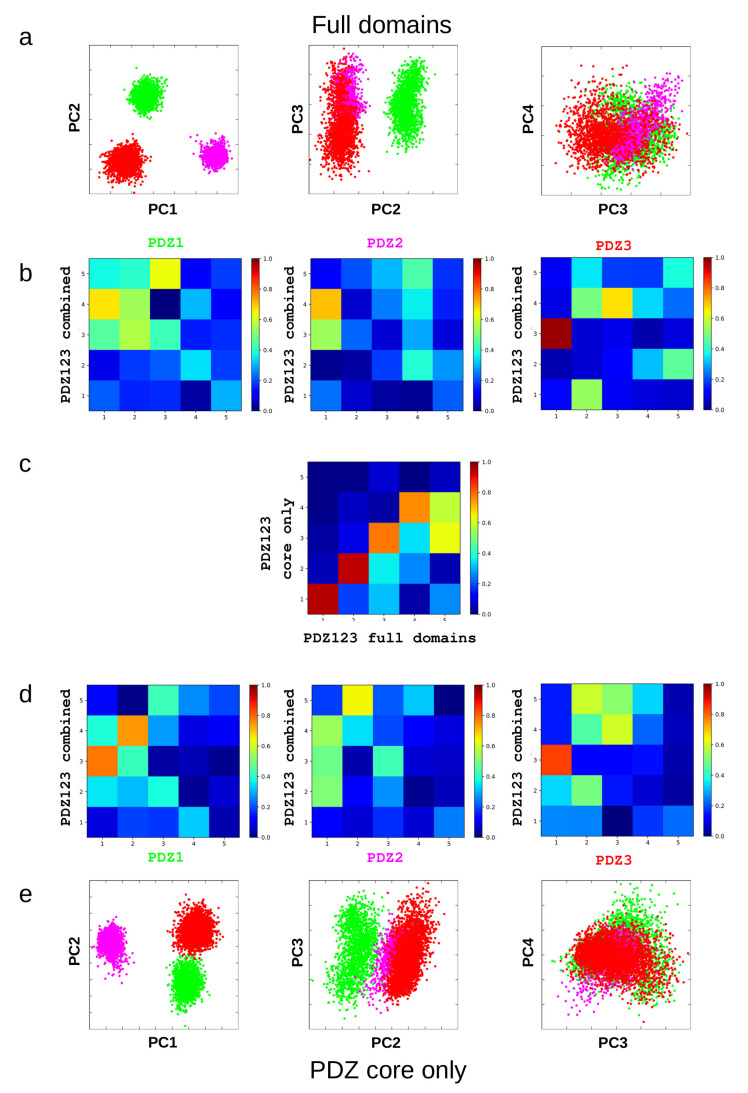
Comparison of dynamic structural ensembles of the three PDZ domains of PSD-95. (**a**) distribution of the PDZ1 (green), PDZ2 (magenta) and PDZ3 (red) ensembles along the PCs 1–2, 2–3 and 3–4 (from left to right, respectively) as well as (**b**) the overlap of PCs calculated for individual ensembles with the combined one. (**d**,**e**) the same as (**a**,**b**) but only using the residues common to all PDZ domains (“PDZ core”). (**c**) comparison of the results obtained with the two analyses showing the overlap for the first 5 PCs.

**Figure 7:**
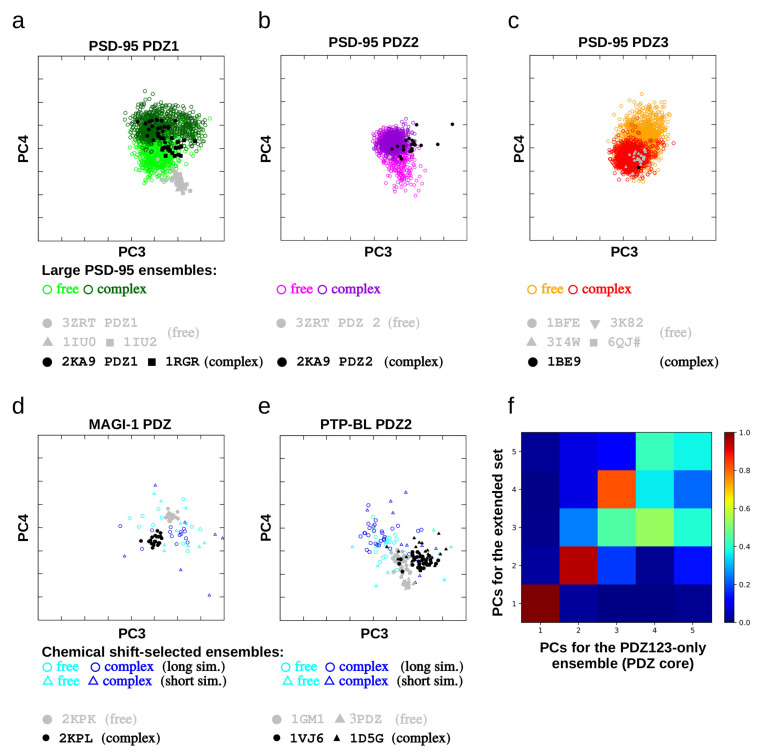
Comparison of the free and bound conformations of selected PDZ domains in the PCA generated for the extended PDZ set. On each plot from (**a**–**e**) the same data points are shown as in Figure 5 but only for selected domains and conformers. (**a**–**c**) PDZ domains 1–3 of PSD-95, respectively. The dynamic ensembles are shown along with some relevant PDB structures. (**d**,**e**) ensembles for the MAGI-1 PDZ and PTP-BL PDZ2 domains selected on the basis of chemical shift data from short and long simulations (see Methods), along with corresponding PDB structures. (**f**) overlap of PCs calculated for the PSD-95 PDZ domains only (same analysis as in Figure 6) and for the extended domain set (shown in Figure 5).

The free and bound states are separated along the same mode, PC4 in the PCA for the extended domain set. This mode largely matches PC3 of the analysis containing only the PSD-95 PDZ domains, where this mode, in turn, reflects PC1, the opening-closing motion, identified in the analysis of PDZ3 ensembles (Table 3 and Figure 3, Figure 6 and Figure 7). For the PSD-95 ensembles, this means that the conformational space covered by the bound, complexed state lies largely within the region spanned by the free state, and this differential distribution occurs along PC4. Surprisingly, the bound states in different PDZ domains populate opposite regions along PC4. This is also observed for ensembles generated with chemical shift-based selection for two other PDZ domains and also largely by the original PDB structures in the sense that their relative order along this motion is consistent with that observed for the ensembles (Figure 7). For PSD-95 PDZ3 we also used the structures recently published by Camara-Artigas et al. [28] and these also conform with our observations but for two D332P mutant structures (PDB ID 6QJF). The exception is the PDB structure pair 1GM1-1VJ6, corresponding to the second PDZ domain of PTP-BL, where the original PDB-deposited ensembles are separated only along PC3. We note that although the chemical shift-selected ensembles from the long simulations do not exhibit better correspondence to the experimental chemical shifts than those selected from then short simulations, they are larger and their conformational space in the PC3-PC4 dimensions is closer to the original NMR structures.

## 3. Discussion

### 3.1. The PDZ3 Ensembles as Models to Describe Internal Motions

Our ensembles were generated with backbone and side-chain S${}^{2}$ order parameter restraints that are not uniformly available for each of the four states. This restraining scheme generally assumes that the structures fluctuate around a well-defined average state. In our calculations no NOE restraints were used that generally confine the sampled conformations to be close to the experimentally determined state. In addition, S${}^{2}$ restraining primarily restricts the directions and not necessarily the amplitude of motions at given sites [39]. Therefore, our ensembles might represent motions somewhat scaled up relative to the actual ps-ns dynamics of the PDZ3 domain. For example, the free Δ7ct ensemble exhibits a bimodal distribution along PC1, and the opening-closing motion might be expected to occur on a slower time scale than specifically reported by S${}^{2}$ order parameters. It should nevertheless be noted that such loop motions might not be unambiguously separated to nonoverlapping, distinct time scales. For example, in the second PDZ domain of HPTP1, loop rigidification upon ligand binding was observed at both the ps-ns and the μs-ms time scale in the β2-β3 loop [40] In summary, we argue that our models provide a representation of the conformational space of the PDZ3 domain that is reasonably consistent with experimentally determined dynamical behavior and is suitable to identify the main types of structural transitions. However, the limitations in our approach, as in any molecular dynamics study, allow that the actual conformational motions might have somewhat different amplitudes and distribution.

### 3.2. PDZ3 Internal Motions and Their Modulation

An interpretation of our observations can be that the presence of helix α3 shifts the conformational equilibrium towards the wide state. This, in turn, affects the opening-closing motion, especially in the absence of the ligand, whereas ligand binding stabilizes the closed conformation. This explanation is consistent with the one offered by Mostarda et al. [22], namely, that the presence of helix α3 would quench the flexibility of the loops surrounding the binding site. However, our model offers a wider framework with dissecting the different motions and factors and suggests a regulation based primarily on population shift between different states, in line with dynamic models of ligand binding and allostery [41]. Accordingly, the peptide ligand and helix α3 exert an antagonistic effect on the conformational landscape by shifting the dynamic equilibria to different states. Zhang et al. have shown that phosphorylation of Tyr397 in helix α3 reduces ligand binding 4-fold and this was suggested to be consistent with  50% undocking of the helix from the PDZ domain. Thus, C-terminal truncation would mimic the effect of this phosphorylation to some extent. Tyr397 is in close proximity to Ser339, a key member of the residue network identified by contact analysis in sheet β3. Ser339 is also one of the residues exhibiting the largest chemical shift perturbations upon Tyr397 phosphorylation [6]. Our model obtained from the investigation of the full and Δ7CT variants is compatible with the effect of docking helix α3 to the domain as described above and in another study on Tyr397 phosphorylation and α3 helix deletion [24]. Specifically, they found that deleting helix α3 alters the interactions between residues in the β2-β3 loop and the ligand. Our model puts these local interactions in a larger framework of motions identified by principal component analysis; namely, we suggest that the interactions with helix α3 modulate the narrow-wide transition of the domain that involves coupled motions of the β2-β3 loop and helix α2. In turn, this rearrangement influences the extent of the opening-closing motion that is the primary one connected to ligand binding.

In the context of the full PDZ3-SH3-GK supramodule or the full-length PSD-95 molecule, such modulative interactions are expected to be more extensive. A full model of the PDZ3-SH3-GK supramodule based on NMR data suggested that the canonical peptide site and loop β2-β3 of PDZ3 is in weak contact with the SH3 domain [7]. Implications of a possible direct insertion of some linker residues (Leu411 and Met412) into the PDZ peptide binding site further suggest a complex interplay between neighboring structural elements. There are many different attempts in the literature to delineate a residue network in PSD-95 PDZ3—and also in other PDZ domains—responsible for intradomain allosteric communication. Our approach is solely based on the residue–residue contacts observed in the structural ensembles of PDZ3. We note that with the exception of Phe325, all residues in our network are located in the “PDZ core”, identified independently from the network itself (Table S5). It means that the contacts formed by these residues are formed between the structural elements common to most, if not all, PDZ domains rather than in the variable loop regions.

### 3.3. Motions in the Light of Other PDZ Domain Structures

**Figure 8:**
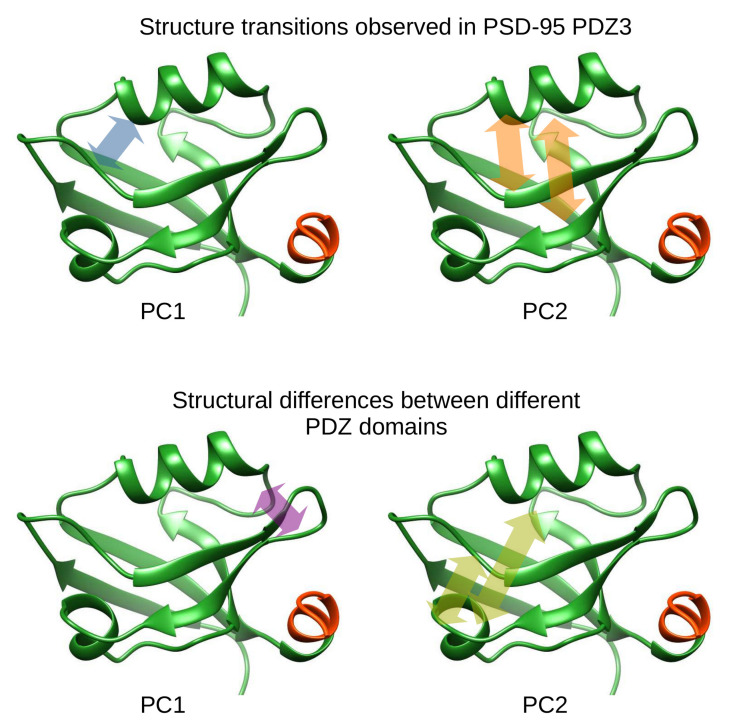
Summary of the most important structural rearrangements/differences within PDZ domains identified in this study. For the structure transitions in PSD-95 PDZ3, PC1 and PC2 refer to the principal components shown in Figure 3 and for the structural differences between domains, PC1 and PC2 refer to the PCA shown in Figure 5.

Our analysis reveals that there is a clear distinction between structural differences between different PDZ domains and the characteristic internal motions (Figure 8). These differences are located in the region connecting strand β6 (PSD-95 PSDZ3 notation) with helix α2, flanking the ligand-binding site opposite to the β2-β3 loop. This observation further reinforces the important role of this loop and the possible modulation of its structure and dynamics by yet another factor besides ligand binding [22], interactions with helix α3 [24] and interdomain interactions [4,7]. The other structural difference is the distance between the α2 helix and the β2 strand. Although this rearrangement seems qualitatively similar to the narrow-wide transition identified for PDZ3, the quantitative overlap between these modes is weak (Figure 6d), partly because of the different movement of the N-terminal part of helix α2 (Figure S5).

Our most unexpected finding was that the free and bound structures are distributed differently with respect to the opening-closing motion for different PDZ domains. For some domains, like PSD-95 PDZ3, the closed form is preferred in the bound state, whereas in PDZ1 and PDZ2, the opposite seems to be the case. The validity of this result is supported by the distribution of the corresponding, deposited PDB structures along this motional mode, which in all cases reflects the order exhibited by the dynamic ensembles. In the case of the chemical shift-selected MAGI-1 and PTP-BL PDZ2 ensembles, the same overall trend can also be observed, albeit the size of these ensembles is much smaller and their diversity is not necessarily directly comparable to those generated by restrained MD.

It also should be noted that when the individual ensembles are analyzed, the conformational differences responsible for the highest variability—as identified by PCA—do not necessarily correspond to the opening-closing motion described here. This observation, most prominently seen for PSD-95 PDZ2, does not falsify our notion that the structures differ along this motional mode, rather, along with the differences in the free and bound states highlighted above, points to the structural and dynamical variability of the PDZ domain family.

### 3.4. Common Motifs and Unique Properties of PDZ Domains Relative to PSD-95 PDZ3

The versatility of PDZ domains with regards to their structure and function has long been recognized. Many domains contain unique extensions that have a role in partner binding. The diversity of partner protein motifs and other molecules is also a well-known phenomenon in this domain family. Our analysis of internal motions in PSD-95 PDZ3 revealed a conformational rearrangement that can be found in practically all PDZ domains investigated, namely, the opening-closing motion of the peptide binding site. This motion affects primarily the β1-β2 loop and the C-terminal part of the α2 helix. This motion seems to be modulated by the narrow-wide transition of the binding site, which is, in turn, modulated by the interactions with the additional α3 helix. Our analysis of the extended domain set suggests that although the opening-closing motion is universal, its redistribution upon ligand binding can be different for distinct domains. Thus, the dynamical aspects of ligand binding, connected to recognition as well as to changes in conformational entropy, can well be another domain-specific feature despite the common architecture and principal motional mode in different PDZ domains.

## 4. Materials and Methods

### 4.1. Datasets and Structures

The starting structures of the simulation were prepared from PDB structures 1BFE and 1BE9, corresponding to the free and complex forms of rat PSD-95 PDZ3 [42]. First, manual modification of the files was performed to match the constructs described in [21], including completion of the side-chain of Asp322, modifying the side-chain of Ile328 to Val and truncating the C-terminus to residue 402 (considered the full-length form, matching that investigated in [21]) or 395 (corresponding to the Δ7CT construct). Missing residues (301–306) at the N-terminus of 1BFE were modeled based on those in 1BE9 for the complex form. The obtained structures were subjected to energy minimization with GROMACS 4.5.5. [43] Backbone and side-chain S${}^{2}$ order parameters for the free and bound states of the full and truncated froms, described in [21] were kindly provided by Dr. Andrew Lee.

### 4.2. Restrained Molecular Dynamics Simulations

Restrained molecular dynamics simulations were performed with an in-house modified version [44] of GROMACS 4.5.5 [43] with S${}^{2}$ restraining implemented [33]. The protocol used was similar to that described in [44], except that no NOE restraints were available. The AMBER99SB force field and TIP4P water model were used. After neutralization and equilibration for 1 ns, 20 ns long ensemble-based simulations were run with 8 parallel replicas for each of the four (free/bound, full-length/truncated) states using the corresponding S${}^{2}$ restraints. The first 10 ns of the simulations were discarded, resulting in 808 conformations for each state. Besides generating ensembles with both backbone and side-chain order parameter restraining, exploratory calculations of 4 ns with only backbone or side-chain S${}^{2}$ restraints were also performed to check the effect of restraint inclusion/omission. As Thr385 of the truncated free form has an unusually low backbone S${}^{2}$ value, control simulations with the omission of a restraint based on this particular parameter were also performed. The differences in the ensembles did not influence the findings and conclusions of the study. Correspondence of the obtained ensembles to experimental order parameters was verified using the CoNSEnsX${}^{+}$ web server [45].

### 4.3. Generation of Small Ensembles with Chemical Shift-Based Selection

We have performed additional molecular dynamics simulations for our extended PDZ set. We have chosen 8 PDB structures (2KPL, 2LC6, 2LC7, 2LOB, 1GM1, 1OZI, 2KPK, 1VJ6, 1UM7) for short simulations using GROMACS 4.5.5 and a protocol similar to the one described above. These unrestrained short simulations were 20 ns long on 8 parallel replicas, with models sampled from 6 ns. From these, 2 pairs of free and complexed structures (PDB models 1GM1—1VJ6 and 2KPK—2KPL) were further analyzed using long, 500-ns single-replica simulations with GROMACS 2020. Here, the AMBER99SB-ILDN force field [46] was used with the spc/e water model and NVT and NPT position-restrained equilibration was included before the production run. Structures were extracted every 10 ps. From both the long and short simulations, subensembles were selected based on Cα and Hα chemical shifts with the CoNSEnsX${}^{+}$ web server [45]. The most important data on these ensembles is summarized in Table S3.

### 4.4. Conformational Entropy Calculations

Conformational entropy of the PDZ3 ensembles was estimated using GROMACS (scripts g_covar and g_anaeig) with backbone as the fit group, only nonhydrogen atoms (group “Protein-H”) and mass weighted approximation. Only atoms found in all systems were considered, thus, the ligand and the C-terminal extension, if present, were omitted to make the results directly comparable. Entropy values were calculated using the quasi harmonic method [36] and the Schlitter formula [37]. The two calculations yielded comparable results and we choose to report the results obtained with the Schlitter formula. We calculated the ratio $\left(S_{cd}-S_{fd}\right)/\left(S_{cf}-S_{ff}\right)$ for the whole ensembles of 808 conformers and also their truncated variants containing gradually more structures to check the robustness/convergence of the value (Figure S2). This ratio was compared to the analogous value obtained from experimental data described in [21].

### 4.5. Analysis of the Ensembles

Principal component analysis of the ensembles was performed with ProDy [47]. Interresidue contact areas were calculated using Voronota [48]. The raw outputs were processed using in-house Perl scripts to calculate the average and standard deviation of each contact in the different states and/or in conformations falling into different regions of the PCA plots. Residue–residue contacts were considered to be discriminative between conformations/states when the difference between the average contact area for the two states is larger than the sum of their respective standard deviations. Basic network analysis of contacts thus selected was done with Cytoscape [49]. Visualization and superposition of the structures were done with MOLMOL [50] and UCSF Chimera [51].

### 4.6. Comparative Analysis of a Large Set of PDZ Domains/Ensembles

PDZ domains selected for the study were chosen after a literature survey. Two NOE-based ensembles of PSD-95 PDZ 1 and 2, published previously by our group [4], were included in the analysis. In addition, small ensembles were generated for a set of PDZ domains with available chemical shifts. The ensembles were selected using the CoNSEnsX${}^{+}$ service for they correspondence to Hα and Cα chemical shifts from a pool of conformers generated by unrestrained MD runs. To define a common core that can be used in a comparative analysis, a multiple structure alignment of all included PDZ domains was created using MAMMOTH-Mult [52]. For this, the first model from NMR structures and a single chain with the PDZ domain from multichain entries was selected. Structures containing tandem PDZ domains were split and the two domains were handled separately. To account for a recently described structural variability in PDZ3 domain variants, we have included all PDZ3 structures reported in [28]. As MAMMOTH-Mult cannot handle all 168 structures in a single run, multiple rounds of alignments were generated and then the resulting alignments were concatenated using a structure common to all alignments. The alignment was converted to a mapping of the original residue numbering in the input PDB files and the residues common to the investigated domains, referred to as the “PDZ core”. Table S4 contains mapping information for PSD-95 PDZ3 and the full MAMMOTH-Mult alignment is provided as a supplementary file in FASTA format (Data set S1). Using this mapping, all PDB structures and ensembles were converted to contain only the core residues. For the PCA analysis, all models in the NMR ensembles were considered. All steps were performed with in-house Perl and Python scripts similar to those used for the analysis described earlier for parvulins [39].

## 5. Conclusions

To our knowledge, our analysis of PSD-95 PDZ3 differs from previous ones in several important aspects. First, it is based on experimentally determined mobility data, namely, backbone and side-chain S${}^{2}$ order parameters from NMR relaxation measurements. Thus, our ensembles reflect experimentally observed dynamics better than those obtained from unrestrained (“conventional”) molecular dynamics calculations. Second, we apply principal component analysis on the ensemble combined for the four states and focus on the internal parts of the domain, discarding flexible tail regions. Our approach makes it possible to detect subtler but functionally important and generally occurring structural transitions. Third, to identify residue networks, we use direct contact analysis and identify residue pairs for which a significant change occurs between the states identified by the principal component analysis. Last but not least, we put these observations into the context of other PDZ domain ensembles and PDB structures by defining a common set of residues, the PDZ core.

We have identified the opening-closing motion in PDZ3 that corresponds to the displacement of helix α and loop β1-β2 and is most influenced by ligand binding. Another motion, the narrow-wide transition, affecting the distance between helix α2 and strand β2, is modulated by the presence of the additional helix α3. Our analysis suggests that the capability of structures to explore regions along the opening-closing motion is dependent on their state along the narrow-wide transition, offering a population shift-based interpretation of dynamic allostery in the domain.

We have identified a network of residues whose physical contacts change upon these structural transitions and span the entire width of the domain along the ligand biding site. These residues connect the site of interaction of helix α3 with the structural elements identified in the motions described above.

We have shown that in a large set of PDZ domains, interdomain structural diversity can be separated from intradomain motions. Analysis of PDZ ensembles reveal the presence of the opening-closing transition in different PDZ domains but also suggests that the redistribution of conformations along this motion is not uniform in different PDZ domains, representing yet another aspect of diversity besides known structural and functional divergence in PDZ domains.

## Figures and Tables

**Table 1:** Correlation of backbone and side-chain S${}^{2}$ values in the calculated PDZ3 ensembles.

Ensemble	Backbone S${}^{2}$	Side-Chain S${}^{2}$
Free full-length (ff)	0.92	0.95
Free Δ7CT (fd)	0.87	0.96
Complex full-length (cf)	0.95	0.97
Complex Δ7CT (cd)	0.83	0.95

**Table 2:** Distribution of the conformers in the individual ensembles according to the principal components. The thresholds between open-closed and narrow-wide conformations are chosen to be 0 along the first and second PCs, respectively. The co-occurrence values provide a measure of how the different states appear simultaneously in the different ensembles.

				Complex	Complex	Free	Free
Ensemble	All4	Δ7CT	Complex	Δ7CT	Full	Δ7CT	Full
Open	53%	35%	10%	2%	18%	69%	97%
Narrow	54%	30%	50%	31%	69%	30%	87%
Open-Narrow co-occurrence	0.56	0.51	0.54	0.68	0.61	0.67	0.85

**Table 3:** Correspondence of selected principal components identified for the extended PDZ set in other PDZ sets analyzed in this study.

PC#	Description	PSD95 PDZ Domains	PSD-95 PDZ3 Only
1	β4-α2 loop dominant	1	-
2	narrow-wide like	2	-
3	α2 seesaw	-	-
4	opening-closing	3	1

## Supplementary Materials

The following are available online at https://www.mdpi.com/1422-0067/21/21/8348/s1, Figure S1: Exploratory PSD-95 PDZ3 ensembles (8x4ns simulation time) generated for this study and their PCA analysis. Figure S2: Calculated ratio of estimated entropy changes for PDZ3 using ensembles of different size. Figure S3: Additional details of the PSD-95 PDZ3 principal component analysis. Figure S4: Principal component analysis of PSD-95 PDZ3 ensembles combined with recently publishes X-ray structures of the domain. Figure S5: Amplitude plots of the movements along PC2 for the individual PDZ3 domain and the extended PDZ set. Table S1: Correlation of backbone and side-chain S2 order parameters as a function of restraints used. Table S2: Back-calculated backbone and side-chain S2 order parameters for the 20 ns ensembles of PSD-95 PDZ3. Table S3: Summary of the ensembles selected based on chemical shifts. Table S4: Residues of PSD-95 PDZ3 used in the comparisons. Table S5: Residue networks in PSD-95 PDZ3 identified in different studies, Data set S1: Structure-based alignment of the PDZ domains used in the study.

## Abbreviations

The following abbreviations are used in this manuscript:

MD	molecular dynamics
PSD-95	Postsynaptic Density Protein-95
PC	Principal component
PCA	Principal component analysis
