# Supplementary material for: Ensemble-Based Analysis of the Dynamic Allostery in the PSD-95 PDZ3 Domain in Relation to the General Variability of PDZ Structures
Source: Int J Mol Sci. 2020 Nov 6;21(21):8348. doi: 10.3390/ijms21218348 (PMC7672539; doi:10.3390/ijms21218348)
Supplement: Supplementary file 1 [file ijms-21-08348-s001.zip › Dudola_SupMat_rev1.pdf]

Supplementary material for

Ensemble-based analysis of the dynamic allostery in the PSD-95 PDZ3 domain in relation to the general variability of PDZ structures

Dániel Dudola, Anett Hinsenkamp, Zoltán Gáspári

Supplementary Figures S1-S5

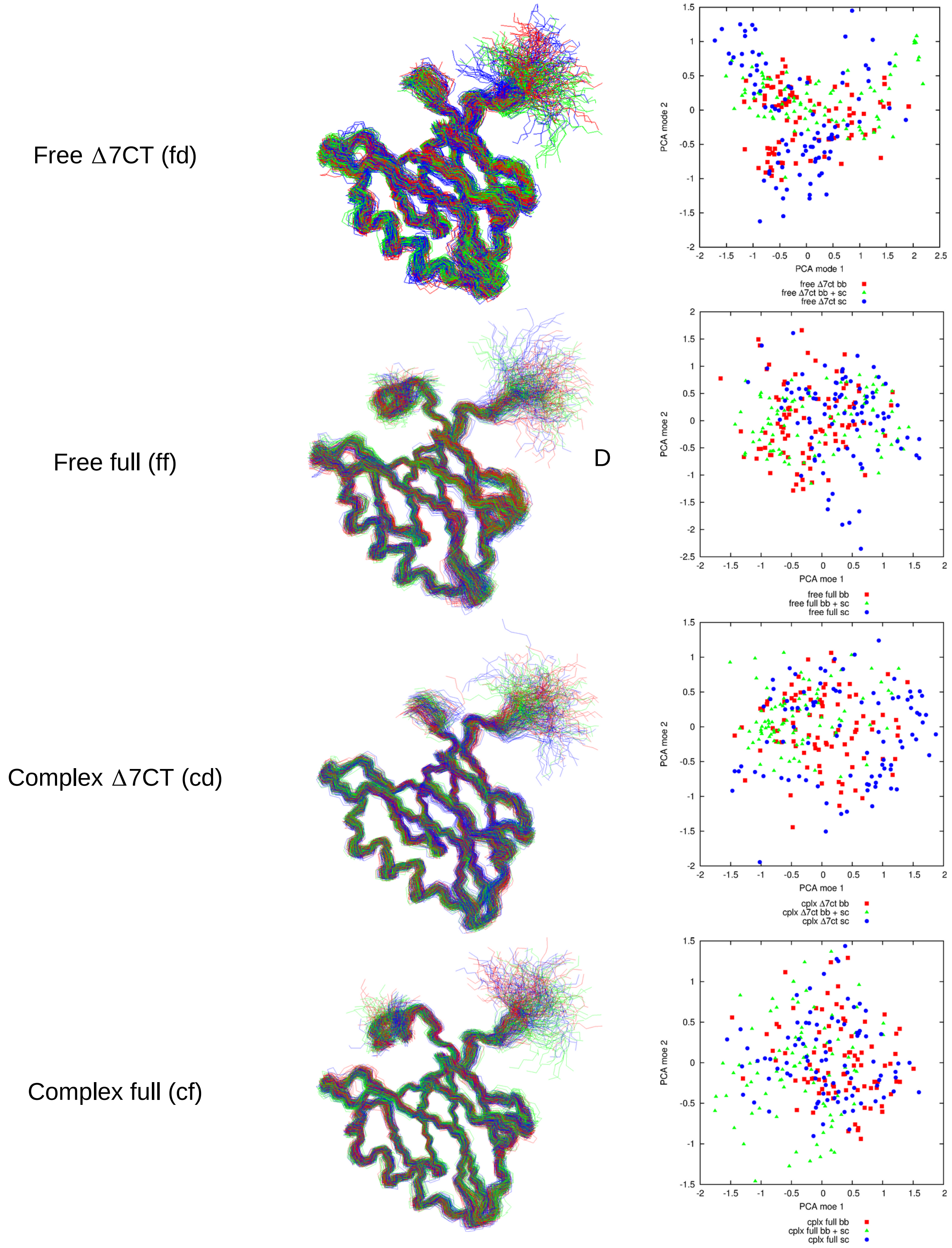

Figure S1. Exploratory PSD-95 PDZ3 ensembles (8x4ns simulation time) generated for this study and their PCA analysis. Red: genaretd with backbone S^2 restraints only, blue: side-chain S^2 restraints only, green: backbone +side-chain S^2 restrains.

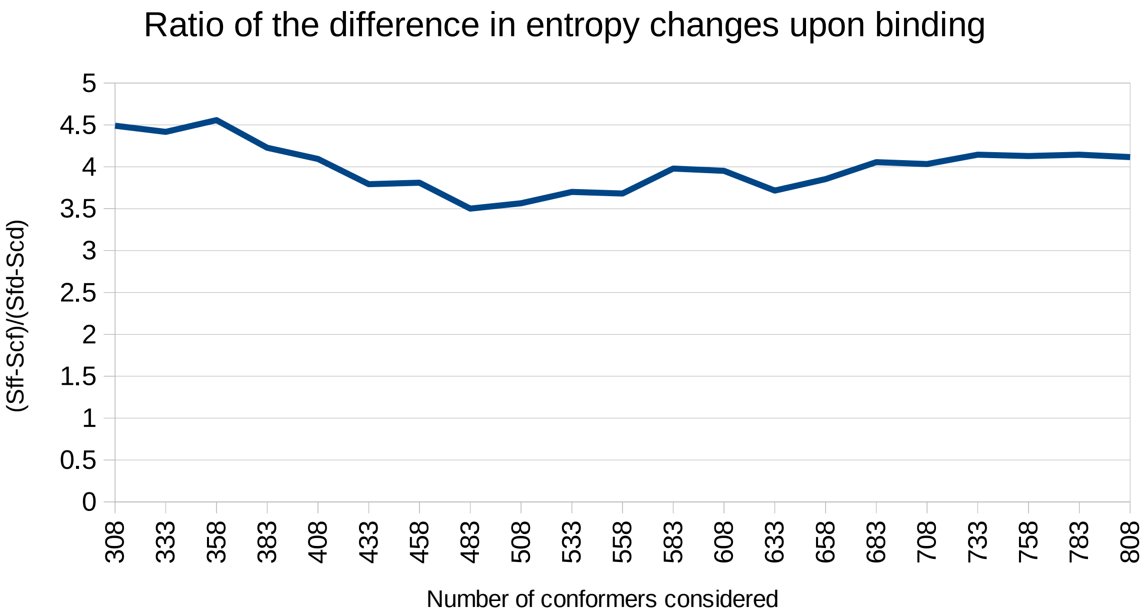

Figure S2. Calculated ratio of estimated entropy changes for PDZ3 using ensembles of different size (sub-ensembles of the full 808-member PDZ3 ensembles)

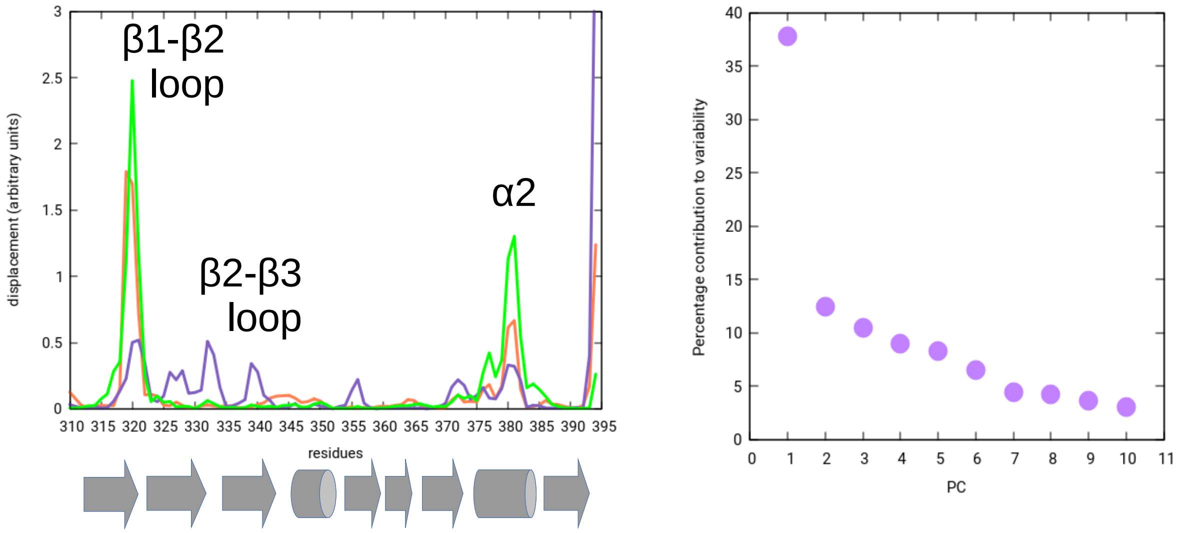

Figure S3. Additional details of the PSD-95 PDZ3 principal component analysis. Left: Cα displacements in the first 3 PC modes. Displacements in modes 2 and 3 are scaled up for better visibility and their ratio to each other and mode 1 is arbitrary. The plots are truncated at the displacement values of the terminal regions. Right: Contribution of each mode to the structural variability of the ensemble.

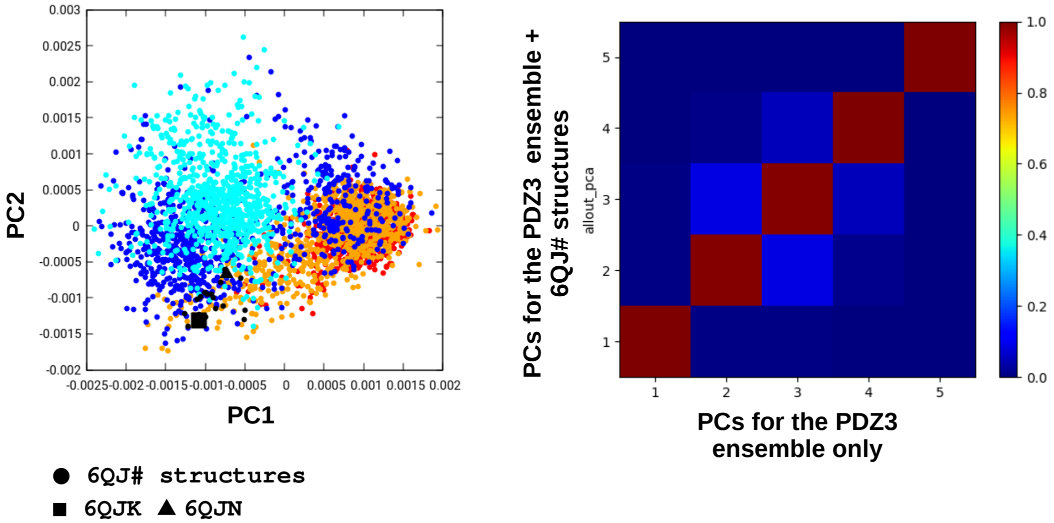

Figure S4. Principal component analysis of PSD-95 PDZ3 ensembles combined with recently publishes X-ray structures of the domain. Left: PCA plot of the PDZ3 ensemble (all 4 states as shown in Figure 3 with the same coloring) combined with the recently published X-ray structures 6QJ#. Two structures with different loop conformations (CI and CII) discussed in Camara-Artigas et al. 2019. are highlighted in blue. Right: PCA overlap diagram showing that the inclusion of the 6QJ# structures does not affect the principal components derived for the PDZ3 ensemble. Modes 1-5 show a very high overlap when calculated with and with the inclusion of these structures, thus, the modes presented in Figure 3 are valid for the combined ensemble. It should be noted that the reasson for the slight difference between this plot and that shown in Figure 3 is that the combined ensembe contains only 83 common residues. To account for β2-β3 loop motions, from all 6QJ# chains only those containing CA coordinates for residue 332 were selected.

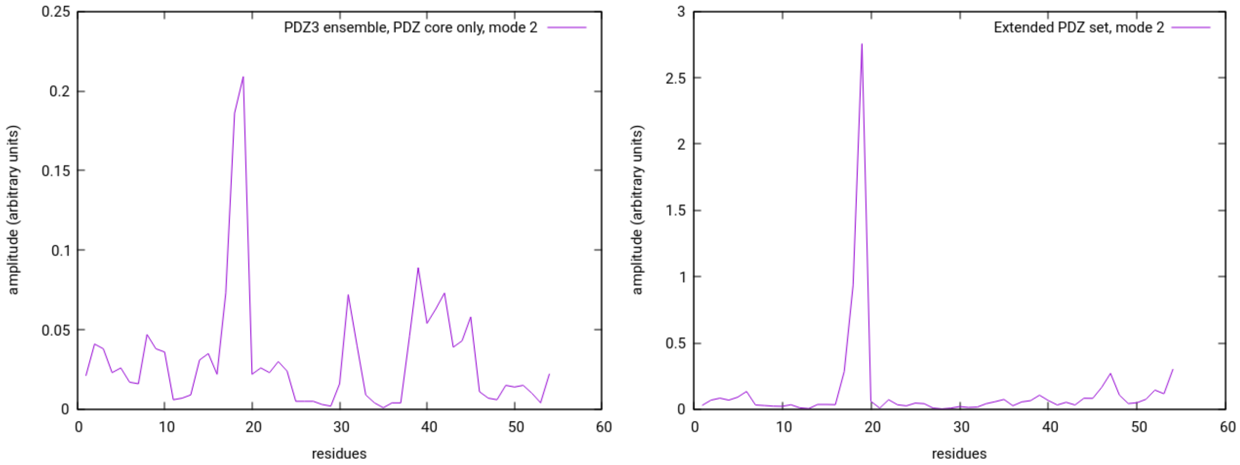

Figure S5. Amplitude plots of the movements along PC2 for the individual PDZ3 domain (PDZ core only, left) and the extended PDZ set (right). PC2 in the extended set is qualitatively similar to the narrow-wide transition identified for PDZ3 (PC2) but the affected regions are somewhat different. The peak around residue 30 corresponds to the β4-β5 loop and the region from ~39-46 to the N-terminal part of helix α2.
